# Examining differences in time to appointment and no-show rates between rural telehealth users and non-users

**DOI:** 10.3389/fdgth.2024.1264893

**Published:** 2024-01-26

**Authors:** Kristin Pullyblank, Nicole Krupa, Melissa Scribani, Amanda Chapman, Megan Kern, Wendy Brunner

**Affiliations:** Bassett Medical Center, Cooperstown, NY, United States

**Keywords:** telehealth, health equity, access to care, rural population, disparities in care

## Abstract

**Background:**

Telehealth has undergone widespread implementation since 2020 and is considered an invaluable tool to improve access to healthcare, particularly in rural areas. However, telehealth's applicability may be limited for certain populations including those who live in rural, medically underserved communities. While broadband access is a recognized barrier, other important factors including age and education influence a person's ability or preference to engage with telehealth via video telehealth or a patient portal. It remains unclear the degree to which these digital technologies lead to disparities in access to care.

**Purpose:**

The purpose of this analysis is to determine if access to healthcare differs for telehealth users compared with non-users.

**Methods:**

Using electronic health record data, we evaluated differences in “time to appointment” and “no-show rates” between telehealth users and non-users within an integrated healthcare network between August 2021 and January 2022. We limited analysis to patient visits in endocrinology or outpatient behavioral health departments. We analyzed new patients and established patients separately.

**Results:**

Telehealth visits were associated with shorter time to appointment for new and established patients in endocrinology and established patients in behavioral health, as well as with lower no-show rates for established patients in both departments.

**Conclusions:**

The findings suggest that those who are unwilling or unable to engage with telehealth may have more difficulty accessing timely care.

## Introduction

The COVID-19 pandemic has prompted increasing interest in studying access to and utilization of telehealth technologies. As healthcare systems rapidly pivoted many of their service lines to telehealth, the health equity implications of the digital divide became clear (1–4). In light of the digital divide, there have been national calls to action for universal broadband access; to ensure access to the hardware and software required to use telehealth, including necessary adaptations for those who may have cognitive, sensory and/or motor impairments which make the current technology difficult to use; to advocate for payment parity; and to provide digital health literacy training as necessary (5–7). Despite these efforts, there has been limited research on how *access to care* is affected through the use of telehealth. We are defining telehealth as a healthcare visit that occurs remotely either through phone or video.

**Figure 1:**
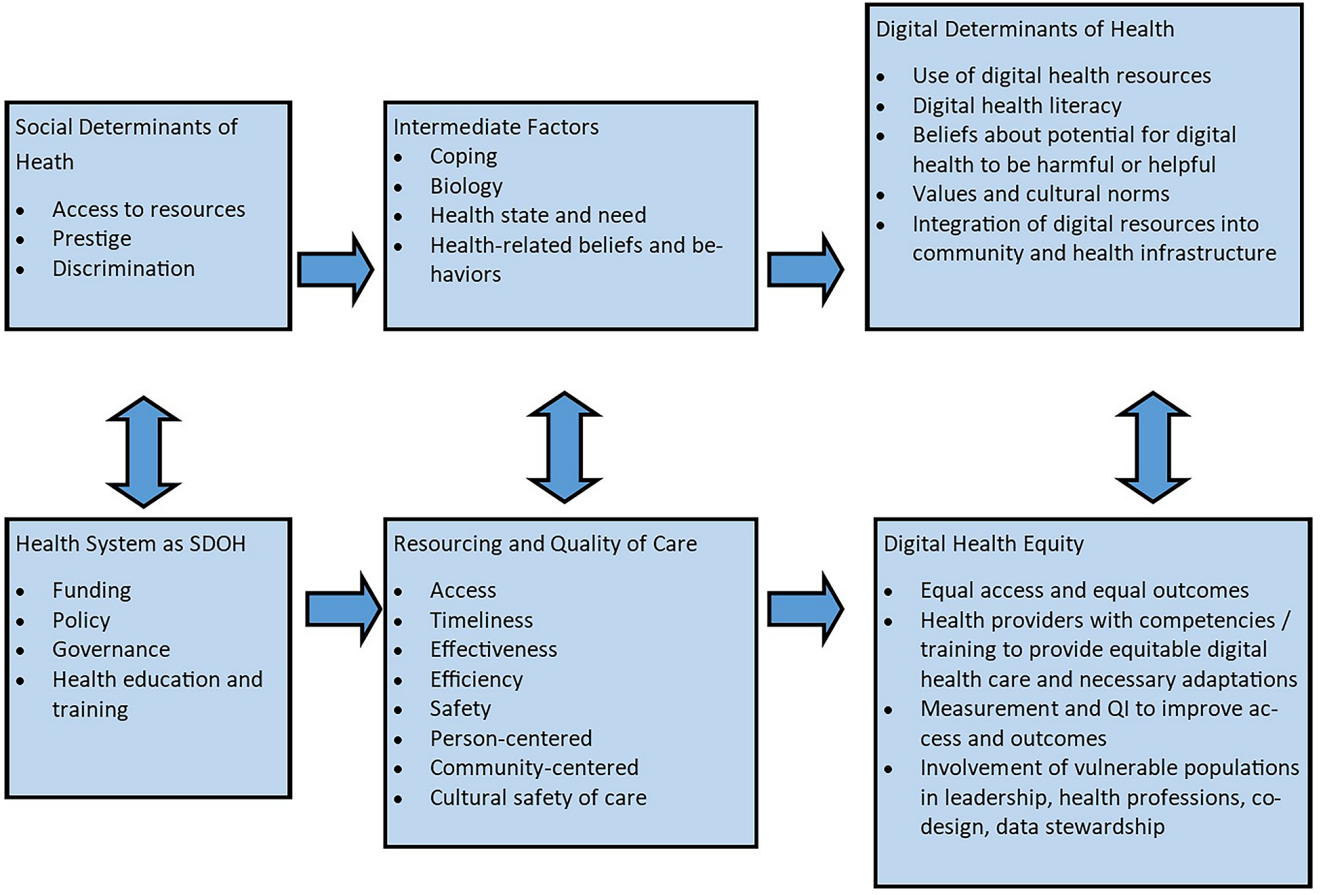
Digital health equity framework adapted from Crawford and Serhal (2020).

Several frameworks are useful for studying access to care in terms of telehealth use. Penchansky and Thomas delineated five different dimensions of access: availability, accessibility, accommodation, affordability, and acceptability (8). As Sieck et al. described, this model can be reconceptualized and applied to telehealth equity (9). In their proposed digital health equity framework (Figure 1), which is based on Dover and Belon's health equity measurement framework (10), Crawford and Serhal consider the complex ecological relationships among a variety of determinants (11). This comprehensive non-linear model describes how social, economic, and cultural factors of both the user and the healthcare system influence the digital determinants of health and subsequently digital health equity and access to care. In this model, digital health equity is predicated, in part, on resourcing and quality of care factors including access, timeliness, effectiveness, cultural and personal safety, person-centeredness, and community-centeredness. In turn, these resourcing and quality of care indicators influence and are influenced by broader social determinants of health at both the individual consumer and health system levels. While numerous studies have described the various determinants that affect access to telehealth, there has been less emphasis on how the ability or willingness to engage in telehealth affects access to care, particularly in rural communities. Our study attempts to address this gap by examining the access indicators of “time to appointment” and “no-show rate” between telehealth users and non-users throughout our rural integrated healthcare system within two specialty departments. We hypothesized that time to appointment would be shorter and no-show rates would be lower among those who used telehealth vs. those who did not use telehealth. It is important to note that we were not seeking to understand how decisions were being made as to whether the appointment would occur via telehealth, but only what happens relative to access when a person does use telehealth vs. not.

## Methods

### Setting

**Figure 2:**
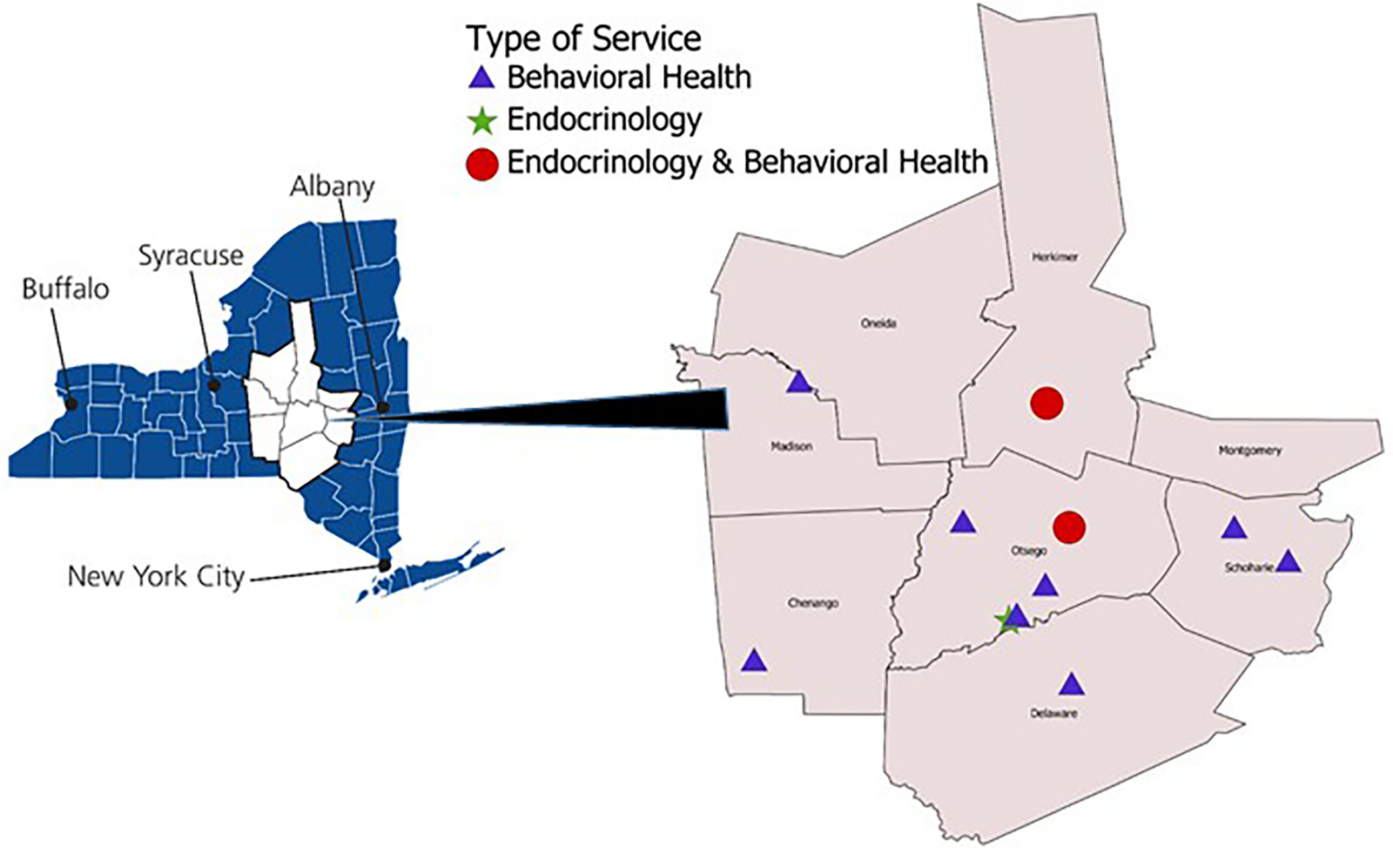
Map of study region.

This retrospective analysis took place in rural upstate New York within a regional, integrated healthcare network. The service area covers 5,600 square miles and consists of six affiliated hospitals, over twenty community health centers, rehabilitation and nursing facilities, and 22 school-based health centers. While there are primary care centers in many of these communities, access to specialty services is limited to just a few sites throughout the network (Figure 2). All counties within the network are considered rural by the Federal Office of Rural Health Policy (FORHP). Within the 8 county area, between 85% and 95% of the population has access to broadband internet, which is lower than the New York State average of 97.4% (12). In addition, between 10% and 20% of the population in these counties do not have an internet subscription, which is higher than the New York State average of 9.7% (13). Our previous research illustrates that having broadband does not equate to a willingness or ability to engage in telehealth (14).

### Data sources

The data source for this analysis was the healthcare network's electronic health record (EHR) data (both encounter data and billing data). Using billing data in addition to encounter data allowed us to address challenges with distinguishing between video and audio-only (i.e., telephone) visits. In order to reduce potential confounders that could impact measures of engagement with the healthcare system, we limited the analysis to two service lines: endocrinology and outpatient behavioral health, two departments with very different patterns of telehealth use. Since the onset of the pandemic, most endocrinology patients are seen in-person, whereas most behavioral health patients are seen remotely. The time period for the analysis was August 1, 2021 through January 31, 2022, when the healthcare organization had settled into a “new normal” of the COVID era.

### Outcomes of interest

The metrics we used as indicators of access to care were *time to appointment* (days) and *no-show rate. Time to appointment* was defined as the number of elapsed days between scheduling and appointment day for the first appointment within the study period in the analysis of time to appointment. *No-show rate* was defined as the number of patients whose first appointment scheduled during the study period was not attended divided by the total number of patients with an appointment scheduled during the study period. This metric does not include those appointments that were canceled or rescheduled ahead of time. We analyzed new patients separately from established patients as telehealth access patterns can be very different based on this factor, thus risking biasing the results (15, 16).

We compared time to appointment and no-show rates by type of visit—i.e., in-person or via telehealth. Telehealth was further categorized when possible as audio-only or videoconference. It was important to delineate audio-only from video as audio-only has demonstrated to be more accessible to populations than video (3, 17). It was not possible to identify type of telehealth (audio-only vs. video) for no-show rates as type of visit was not captured in the EHR billing data for scheduled visits that did not take place. Similarly, in the analysis of time to appointment by type of visit, time to appointment was defined as the number of days elapsed between scheduling and appointment day for the first *completed* appointment over the study period because we were unable to identify the type of visit (in-person vs. telehealth) when the visit did not occur. Appointments that originated at a healthcare clinic and involved a telehealth component (e.g., with a specialist) were excluded from this analysis.

The covariates for this analysis came from the EHR and included patient gender (male/female), age, rurality (where rural is defined by Rural-Urban Commuting Area code ≥7), primary payer for the visit (% Medicaid), and use of the patient portal (yes/no). This study was approved by the Mary Imogene Bassett Hospital Institutional Review Board.

### Statistical analysis

Separate statistical analyses were carried out for the two medical specialties (endocrinology and behavioral health); within each specialty, analyses were stratified by new patient visits and established patient visits. Tests of association between patient characteristics and visit type (in-person vs. telehealth) were carried out using chi-square for dichotomous variables and the Kruskal–Wallis test for age. The initial test of association between visit type and median days to appointment was completed by the Kruskal–Wallis test. Analysis of covariance was used to compare days to appointment across visit types (telehealth vs. in person) controlling for gender, payer, new patient status, age, rurality, portal use/non-use and no-show rate. Due to a non-normal distribution of days to appointment, data were first converted to ranks for adjusted modeling, with *post hoc* pairwise comparisons between in-person, telephone and video visits using Sheffé's adjustment. Analysis of no-show status by visit type was carried out using univariate logistic regression and multiple logistic regression controlled for gender, payer, new patient status, age, rurality, portal use/non-use, and days to appointment.

## Results

### Visit type distributions and associated factors

Between August 2021 and January 2022, there were 2,981 unique patients who completed an outpatient behavioral health visit (Table 1); of these, 481 (16.1%) were new patient visits and 2,500 (83.9%) were return visits for established patients. Among new patient behavioral health visits, 23 (4.8%) completed a remote audio-only (telephone) visit; 276 (57.4%) completed a remote video visit; and 182 (37.8%) completed an in-person visit. Among return behavioral health visits, 462 (18.5%) completed a remote audio-only visit; 1,569 (62.8%) completed a remove video visit; and 469 (18.8%) completed an in-person visit.

**Table 1:** Visit type distributions and associated factors*.

	In-person visit	Audio-only visit	Video-only visit	*p*-values
Total behavioral health visits	651	485	1,845	
New behavioral health visits, *N* (%)	182 (37.84%)	23 (4.78%)	276 (57.38%)	
Age, Median	41^a^	41^a^	36^b^	0.0007
Gender (% female)	66.48^a,b^	47.83^a^	70.65^b^	0.0677
Payer (% Medicaid)	34.62^a^	73.91^b^	40.22^a^	0.0013
Rural, %	38.46^a^	69.57^b^	51.45^b^	0.0023
Active portal users, %	70.88^a^	60.87^a^	89.86^b^	<0.0001
Follow-up behavioral health visits, *N* (%)	469 (18.76%)	462 (18.48%)	1,569 (62.76%)	
Age, Median	52^a^	56^b^	38^c^	<0.0001
Gender (% female)	67.59^a^	70.35^a^	70.43^a^	0.4871
Payer (% Medicaid)	28.14^a,b^	27.27^a^	32.95^b^	0.0226
Rural, %	42.43^a^	52.81^b^	46.97^a^	0.0062
Active portal users, %	75.69^a^	69.26^b^	91.46^c^	<0.0001
Total endocrinology visits	3,667	99	183	
New endocrinology visits, *N* (%)	979 (94.96%)	13 (1.26%)	39 (3.78%)	
Age, Median	59^a^	61^a^	46^b^	0.0042
Gender (% female)	62.41^a^	69.23^a^	76.92^a^	0.1647
Payer (% Medicaid)	21.04^a^	15.38^a^	23.08^a^	0.8405
Rural, %	41.27^a^	41.67^a,b^	58.97^b^	0.0894
Active portal users, %	66.29^a^	69.23^a^	97.44^b^	0.0002
Follow-up endocrinology visits, *N* (%)	2,688 (92.12%)	86 (2.95%)	144 (4.93%)	
Age, Median	62^a^	61^a^	49^b^	<0.0001
Gender (% female)	59.41^a^	68.60^a,b^	73.61^b^	0.0009
Payer (% Medicaid)	16.33^a^	20.93^a^	22.22^a^	0.1051
Rural, %	46.58^a^	48.84^a^	47.92^a^	0.8783
Active portal users, %	68.94^a^	72.09^a^	94.44^b^	<0.0001

* Values across a row not sharing any superscript letter are significantly different by Sheffé's test at *p* < 0.05.

Considering new patient behavioral health visits, utilization of in-person, video and audio-only visits differed across age, gender, payer, rural residence and patient portal use. Specifically, the median age was significantly younger among those completing a remote video visit compared to audio-only or in-person visit types. Females were more likely to complete a video-only visit than an audio-only visit, and those with Medicaid were significantly more likely to complete an audio-only visit than video or in-person visit. Rural residence was associated with a higher likelihood of an audio-only or video visit compared to an in-person visit. Those with an active patient portal account were more likely to complete a video visit compared to an in-person or audio-only visit.

For return behavioral health visits, the type of visit completed did not differ by gender, but did differ for age, payer, rural residence and patient portal use. Those completing video visits were significantly younger than those completing audio-only or in-person visits, and those with Medicaid were more likely to have a video visit compared to an audio-only visit. Rural residence was associated with a greater likelihood of an audio-only visit compared to in-person or video-only visits. Those with an active patient portal account were more likely to have a video visit compared to an in-person visit or audio-only visit; however, active portal users were more likely to have an in-person visit than an audio-only visit.

There were 3,949 unique patients who completed an outpatient endocrinology visit (Table 1); of these, 1,031 (26.1%) were new patient visits and 2,918 (73.9%) were return visits for established patients. Among new patient endocrinology visits, 13 (1.3%) completed a remote audio-only visit; 39 (3.8%) patients completed a remote video visit; and 979 (95.0%) patients completed in-person visits. Among return endocrinology visits; 86 (3.0%) completed a remote audio-only visit; 144 (4.9%) completed a remote video visit; and 2,688 (92.1%) completed an in-person visit.

Considering new patient endocrinology visits, those completing video visits were younger than those completing audio-only or in-person visits. Those with active patient portal accounts were more likely to have a video visit compared to in-person or audio-only visit. Type of visit did not differ by gender, Medicaid payer or rural residence.

For return endocrinology visits, those completing a video visit were younger than those completing an in-person or audio-only visit. Females were more likely to participate in a video visit than an in-person visit. Those with an active patient portal account were significantly more likely to have a video visit compared to in-person or audio-only visit. Medicaid payer and rural residence were not associated with remote vs. in-person visit types.

### Time to appointment

There were statistically significant differences in time to appointment by type of visit for both service lines (Table 2). For outpatient behavioral health new patient visits, median time to appointment was significantly longer for video visits at 31 days compared to in-person (26 days) or audio-only visits (20 days). This difference remained statistically significant after controlling for age, gender, Medicaid payer, rural residence and active portal use. For return outpatient behavioral health visits, the median time to appointment was statistically significantly longer for in-person (28 days) vs. audio-only (27 days) and video visits (24 days).

**Table 2:** Time to appointment (days) by type of visit*.

	In-person visit	Audio-only visit	Video-only visit	*p*-value
New behavioral health visits				
Days to appointment, Median (IQR), Range	25.5 (11–37)^a^ 0–169	20 (12–33)^a^ 0–59	31 (17.5–42.5)^b^ 0–201	0.0018
Follow-up behavioral health visits				
Days to appointment, Median (IQR), Range	28 (13–46)^b^ 0–165	27 (8–42)^a^ 0–112	24 (8–36)^a^ 0–161	<0.0001
New endocrinology visits				
Days to appointment, Median (IQR), Range	37 (20–61)^a^ 0–365	47 (21–73)^a,b^ 1–154	19 (3–63)^b^ 0–199	0.0374
Follow-up endocrinology visits				
Days to appointment, Median (IQR), Range	113 (41–184)^b^ 0–377	65.5 (22–179)^a^ 0–365	49 (15–176)^a^ 0–375	<0.0001

* Values across a row not sharing any superscript letter are significantly different by Sheffé's test at *p* < 0.05.

For endocrinology, the median time for a new patient visit was significantly longer for in-person visits at 37 days compared to video visits at 19 days. This difference remained statistically significant after controlling for age, gender, Medicaid payer, rural residence and having an active patient portal account. Among return endocrinology visits, the median time for an in-person visit was significantly longer at 113 days than either video (49 days) or audio-only visits (66 days). This difference again remained significant after controlling for all relevant covariates.

### No-show rates

For new patient visits, no-show rates did not differ significantly between in-person and remote scheduled visits for either outpatient behavioral health or endocrinology (Table 3). However, no-show rates did differ between remote and in-person scheduled return visits for both outpatient behavioral health and endocrinology. For return behavioral health visits, the no-show rate was significantly lower for remote visits compared to in-person visits (11.5% vs. 16.1%, OR = 0.68, *p* = 0.004). For return endocrinology visits, the no-show rate was again significantly lower for remote visits than for in-person visits (3.3% vs. 11.1%, OR = 0.27, *p* < 0.001). For both service lines, these differences remained significant after controlling for age, gender, Medicaid payer, rural residence and patient portal use.

**Table 3:** No show rates and characteristics by type of visit.

	In-person	Remote	*p*-value
New behavioral health visits, *N*	235	283	
No-show, %	10.64	6.36	
Age, Median	39	38	0.0053
Gender (% female)	66.38	67.84	0.7781
Payer (% Medicaid)	37.02	42.40	0.2416
Rural, %	40.85	52.30	0.0104
Active portal users, %	70.21	86.57	<0.0001
Time to appointment, Median	28	31	0.0199
Odds of No-Show vs. in-person (crude)		0.57 (95% CI: 0.30–1.07)	0.0820
Odds of No-Show vs. in-person (adjusted)		0.61 (95% CI: 0.31–1.21)	0.1548
Follow-up behavioral health visits, *N*	573	2,044	
No-show, %	16.06	11.50	
Age, Median	49	41	<0.0001
Gender (% female)	66.49	70.21	0.0907
Payer (% Medicaid)	30.54	32.00	0.5421
Rural, %	42.76	48.63	0.0138
Active portal users, %	75.92	85.91	<0.0001
Time to appointment, Median	28	28	0.0063
Odds of No-Show vs. in-person (crude)		0.68 (95% CI: 0.52–0.88)	0.0037
Odds of No-Show vs. in-person (adjusted)		0.64 (95% CI: 0.49–0.84)	0.0011
New endocrinology visits, *N*	1,125	117	
No-show, %	16.09	10.26	
Age, Median	58	58	0.7237
Gender (% female)	60.98	79.49	<0.0001
Payer (% Medicaid)	23.38	17.95	0.2041
Rural, %	41.10	55.17	0.0041
Active portal users, %	63.47	76.07	0.0061
Time to appointment, Median	39	108	<0.0001
Odds of No-Show vs. in-person (crude)		0.60 (95% CI: 0.32–1.11)	0.1008
Odds of No-Show vs. in-person (adjusted)		0.74 (95% CI: 0.39–1.42)	0.3667
Follow-up endocrinology visits, *N*	2,914	274	
No-show, %	11.05	3.28	<0.0001
Age	61	58	0.0005
Gender (% female)	59.33	68.98	0.0019
Payer (% Medicaid)	17.54	18.98	0.5619
Rural, %	46.43	45.26	0.7515
Active portal users, %	67.57	83.21	<0.0001
Time to appointment, Median	119	98	0.0007
Odds of No-Show vs. in-person (crude)		0.27 (95% CI: 0.14–0.54)	0.0002
Odds of No-Show vs. in-person (adjusted)		0.27 (95% CI: 0.14–0.53)	0.0002

## Discussion

The purpose of this analysis was to determine if there is a relationship between telehealth use and access to care as indicated by time to appointment and no-show rates. Our findings suggest that among those who access care for follow-up appointments via telehealth within the endocrinology or outpatient behavioral health service lines, there is a shorter time to appointment and lower no-show rate than among those who access in-person care within these service lines.

We had hypothesized that appointment wait times would be shorter for telehealth visits than for in-person visits and our results generally supported this hypothesis. Recent research corroborates our findings. A cross-sectional observational study within primary care conducted by Graetz and colleagues prior to the COVID pandemic, found that among patients who scheduled their own appointments through the patient portal (18), there was a significantly shorter time to appointment for telehealth visits vs. in-person visits. Similar to our findings, Graetz et al. found that in-person visits had the longest wait time (19), but in their population, individuals opting for an audio-only visit had the shortest time to appointment, while we found video-enabled appointments had the shortest time to appointment. However, for new behavioral health visits, we found that the longest time to appointment was for video visits, which is contrary to our hypothesis.

There are several possible explanations for the finding that time to appointment is shorter for telehealth visits than for in-person visits among established patients. Older individuals are less likely to use telehealth, and are more likely to have complex health issues. Therefore, the wait to see a specialist (for more complex problems that are more likely to be done in person) may be longer than to see a mid-level provider for a routine check-up (which could be done via telehealth).

Another structural element that may be influencing time to appointment is that clinicians rotate their in-person days among several clinics throughout the region, and thus it becomes more difficult to schedule an in-person visit that is convenient for the patient. In addition, there are providers in both departments who exclusively use telehealth (i.e., are physically located outside of the region). According to scheduling staff, these providers have more flexible schedules making it easier to get patients in. However, it is unclear if this flexibility is due to patients not knowing about the telehealth-only option, patients not being medically appropriate for the services, or personal preference of the patient to use the service.

In addition to patient preference, provider preference may be driving some of the decision to use telehealth. Rodriguez et al. discussed the complexity of who is driving the decision to engage in telehealth or in-person (20). These authors found that practice or clinician factors were responsible for more variance in the decision to use telehealth than patient level factors. While this quantitative analysis was unable to discern practice vs. patient level factors, our previous research indicates that clinicians were often the ones deciding if and when to offer telehealth to any given patient (21). In doing so, there is the risk of implicit clinician bias regarding whether a patient has the capacity to use telehealth, thus resulting in a longer wait time to appointment for that patient. At the same time, we also heard from some providers that using telehealth for visits that were appropriate could open up more in-person appointments for others.

In fact, this last reason may be why getting an in-person appointment was faster in behavioral health for new patients. There is a strong preference among providers to see their new patients in person (22) and by shifting established patients to telehealth, there is theoretically more availability for new patients to be seen in person.

We had also hypothesized that no-show rates would be lower for those using telehealth, which was partially supported by our findings. While no-show rates were significantly lower for telehealth visits (video or audio-only) among established patients, there was no difference for new patients. Kubes et al. found that appointment cancellations among all ambulatory clinic visits were significantly lower among telemedicine relative to in-person appointments (23). They suggest that there are fewer barriers to attending telemedicine appointments. Interestingly, these researchers also found that there was no difference in adverse clinical events between in-person and telemedicine appointments. Alkilany et al. also found that there were significantly fewer cancellations and no-shows for telehealth appointments compared to in-person appointments for visits to a rheumatology clinic (24). Their study period covered the first wave (and most severe lockdowns) of the pandemic. Neither of the aforementioned studies distinguished between new and established patients. Our analysis indicates that even in the post-acute pandemic era, there were still fewer no-shows via telehealth for established patients, supporting the hypothesis that there are fewer barriers to access telehealth appointments.

Other research has found that there is no difference in no-show rates between telehealth users and non-telehealth users (25–27), suggesting that there are other reasons for non-attendance aside from travel barriers, which may be more applicable for new patients, such as not remembering appointment details. An important implication of the finding that there was no difference in no-show rate among new patients who scheduled a telehealth vs. an in-person appointment supports the idea that the same barriers preventing a person from accessing an in-person appointment are present when accessing a telehealth appointment. Consequently, we must question whether telehealth is in fact increasing access to care among those who previously had difficulty in accessing the health care system.

Several limitations must be noted. First, our study occurred within a certain time frame of the peri-pandemic era (mid 2021 to early 2022) and there were a variety of pandemic-related restrictions within the health care facilities that may have affected health seeking behaviors. For example, we recently learned that as of January 2023, our outpatient behavioral health department recommended that new patients be seen in person before allowing telehealth visits, which was not the case during the study period. Second, there may be other more relevant indicators of access to care that were not measured. Finally, in our analysis of the EHR data, we were not able to account for the context of the patient visits. The driving forces behind how decisions are made to receive care through telehealth remain unclear (e.g., provider preference, departmental guidelines, complaint acuity), and it is possible that such factors would be related to the outcomes of time to appointment and no-show rates. Research exploring these factors needs to occur in order to better understand the nuances of telehealth use, access to care and health equity.

## Conclusions

Determining how ability and willingness to use telehealth affects access to care and subsequent health outcomes is inherently complex. Telehealth is not appropriate for all patients and patient visits, and for telehealth originating from the patient's home, its applicability is limited at the very least to non-procedural appointment types. The equity concern arises when access to care is different for those who are able and willing to use telehealth vs. those who are not. We found there were differences in no-show rates and time to appointment based on visit type (video visit, audio-only visit or in-person) as well as patient status (new patient vs. established patient) within the two service lines we studied.

Over the past three years, health systems have been grappling with the question of telehealth equity. It became very clear early on in the pandemic that certain populations were using telehealth more frequently (17, 28–30). The challenge facing healthcare systems today is effectively implementing telehealth technologies so that the intervention does not worsen existing health disparities (31–34). Healthcare organizations must first be willing and able to co-create solutions with their community members to address the upstream factors as outlined in the digital healthy equity framework before the population-level benefits of telehealth can be fully realized.

## Data Availability

The raw data supporting the conclusions of this article will be made available by the authors, without undue reservation.
